# *mer*-Bis(quinoline-2-carboxaldehyde 4-ethyl­thio­semicarbazonato)nickel(II) methanol 0.33-solvate 0.67-hydrate

**DOI:** 10.1107/S2414314624003390

**Published:** 2024-04-26

**Authors:** Raudhatul Nadhirah Awang Adam, Natasha Ann Keasberry, Malai Haniti Sheikh Abdul Hamid

**Affiliations:** a Chemical Sciences, Faculty of Science, Universiti Brunei Darussalam, Jalan Tungku Link, Gadong, BE1410, Brunei; Benemérita Universidad Autónoma de Puebla, México

**Keywords:** crystal structure, nickel(II), quinoline, octa­hedral geometry, meridional coordination

## Abstract

In the title compound, [Ni(C_13_H_13_N_4_S)_2_]·0.33CH_3_OH·0.67H_2_O, the Ni^II^ atom is coordinated by two tridentate quinoline-2-carboxaldehyde 4-ethyl­thio­semi­car­ba­zonate ligands in a distorted octa­hedral shape. At 100 K, the crystal symmetry is monoclinic (space group *P*2_1_/*n*). A mixture of water and methanol crystallizes with the title complex, and one of the ethyl groups in the coordinating ligands is disordered over two positions, with an occupancy ratio of 58:42. There is inter­molecular hydrogen bonding between the solvent mol­ecules and the amine and thiol­ate groups in the ligands. No other significant inter­actions are present in the crystal packing.

## Abstract 2

The title complex exhibits a distorted octa­hedral geometry about the metal centre, which coordinates two tridentate ligands that are perpendicular to each other.

## Graphical abstract

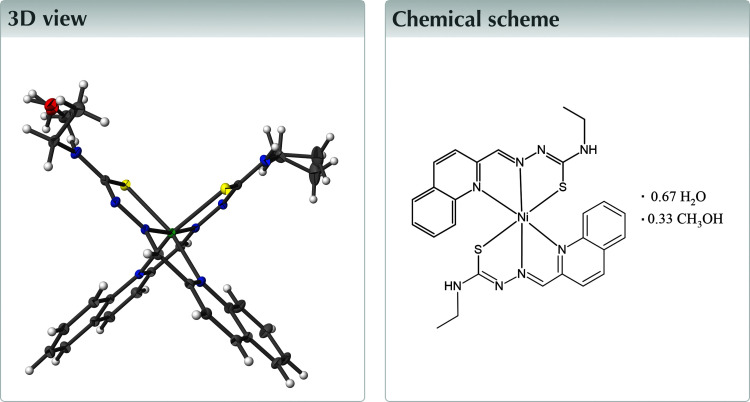

## Structure description

Thio­semicarbazones are a type of Schiff base ligand formed by the condensation of thio­semicarbazides with carbonyl compounds (Arulmurugan *et al.*, 2010 ▸). They com­monly behave as *N*,*S*- or *N*,*N*′,*S*-chelating agents, coordinating the metal through the imine N and S atoms. They frequently feature more than two covalent sites, the number depending on the aldehyde used during the synthesis and on the tautomeric equilibrium of the thio­semicarbazone (Latheef *et al.*, 2021 ▸; Osman *et al.*, 2021 ▸). The versatility of the compounds, along with their metal complexes, have attracted significant inter­est in the fields of chemistry and biology. They exhibit a broad spectrum of biological properties, including anti­bacterial, anti­cancer, anti­proliferative and anti­viral activities (Chaturvedi, 2012 ▸; Damit *et al.*, 2021 ▸; Montalbano *et al.*, 2023 ▸; Kumar *et al.*, 2023 ▸; Arif *et al.*, 2024 ▸). For instance, a series of quinoline-2-­carboxaldehyde thio­semicarbazone derivatives and their Cu^II^ and Ni^II^ complexes have been reported by Bisceglie *et al.* (2015 ▸) for biological survey studies.

Ni^II^ complexes having sulfur donors have been studied, receiving considerable attention due to the identification of a sulfur-rich coordination environment in biologically relevant nickel ompounds, such as the active sites of certain ureases, hydrogenases, as well as de­hydrogenases, that may play a role in the supposed mutagenicity of nickel compounds (Latheef *et al.*, 2021 ▸). The coordination chemistry of nickel is thus of inter­est with respect to its important roles in biological systems (Jayakumar *et al.*, 2022 ▸; Sankar & Sharmila, 2023 ▸). This is due to the ability of nickel to adopt different coord­ination environments, such as tetra­hedral, square planar and octa­hedral. The nickel ion can also bind to soft and hard donor ligands, which allows its coordination chemistry to encompass a variety of coordination environments, coordination numbers and oxidation states (Jayakumar *et al.*, 2022 ▸).

**Figure 1:**
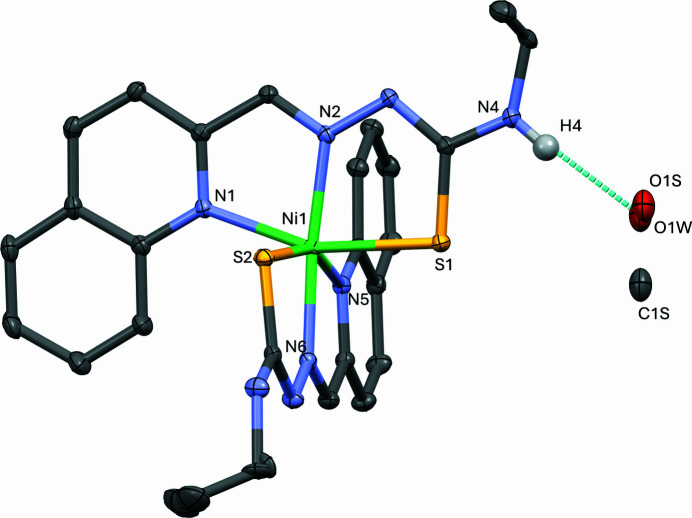
The mol­ecular structure of the title compound with the more abundant ethyl-group positions. Displacement ellipsoids are drawn at the 50% probability level and H atoms have been omitted for clarity (with the exception of H4). Atoms C1*S*, O1*S* and O1*W* are for the disordered solvent molecules, methanol and water. The dashed bond is the inter­molecular hydrogen bond between the complex and the methanol mol­ecule (Table 1 ▸, entry 2).

The asymmetric unit of the title compound contains one complex with formula C_26_H_26_N_8_S_2_Ni and a mixture of water and methanol. The structure of the title compound is confirmed to be in a 2:1 ligand–metal complex, where the two ligands are perpendicular to each other in a distorted octa­hedral shape, coordinating in a *meridional* fashion (Fig. 1 ▸). This aligns with the methyl analogue of the complex found in the literature, which has methyl groups in place of the ethyl groups (Bisceglie *et al.*, 2015 ▸), with its two ligands coordinating as anionic deprotonated mol­ecules. In the structure reported herein, one of the ethyl groups is disordered over two positions, with an occupancy ratio refined as 58:42. The occupancy ratio of the solvent molecules, methanol and water, was refined to 1/3:2/3.

**Figure 2:**
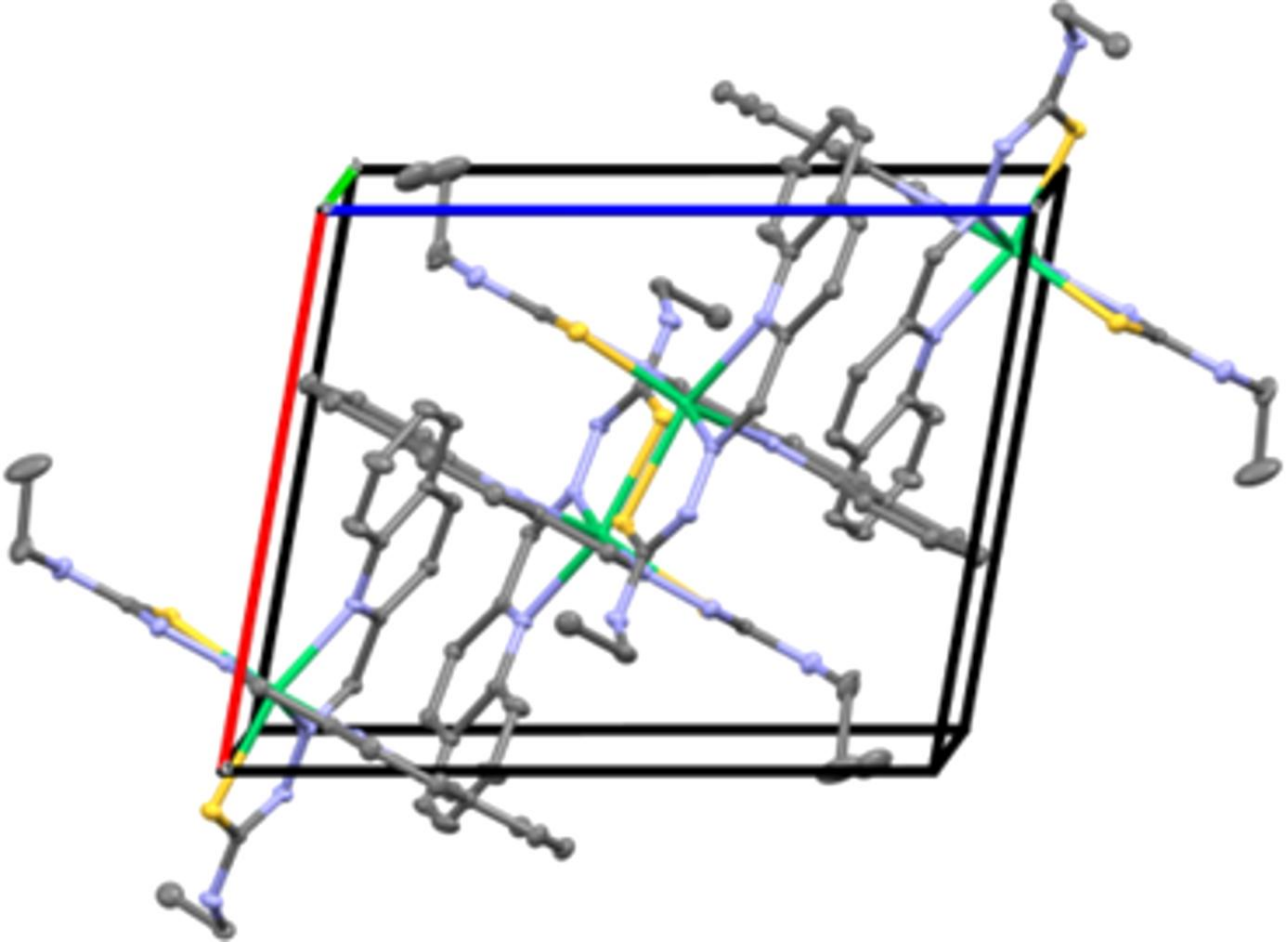
Perspective view of the crystal packing of the title compound approximately along the *b* axis. Solvent mol­ecules and H atoms have been omitted for clarity.

Each methanol/water mol­ecule bridges two neighbouring complexes through inter­molecular N4—H4⋯O(solvent) and OH(solvent)⋯S2^i^ hydrogen bonds (Table 1 ▸). The crystal structure is further stabilized by weaker inter­molecular N8—H8⋯N3^ii^ hydrogen bonds, forming a tri-periodic supra­molecular network (Fig. 2 ▸). No other significant inter­actions are present in the crystal packing of the title compound. In contrast, in the case of the methyl analogue complex, no solvent is present in the unit cell (Bisceglie *et al.*, 2015 ▸). Furthermore, this methyl complex also features inter­molecular inter­actions of C—H groups with the quinoline π-system, which are not observed in the structure reported herein. The Ni—N bond lengths are in good agreement with those observed in other octa­hedral [Ni(*N*,*N*′,*S)*_2_]^2+^ complexes retrieved from the Cambridge Structural Database (CSD, Version 5.45; Groom *et al.*, 2016 ▸): refcodes NOTWEA (Min *et al.*, 2014 ▸), and JUKRAK and JUKQUD (Bisceglie *et al.*, 2015 ▸). To the best of our knowledge, no quinoline-2-carboxaldehyde 4-ethyl­thio­semicarbazonate–nickel(II) complexes have been deposited in the CSD so far.

In the title compound, no H peaks were located on N3 and N7 in a difference map. Additionally, the C11=N3 and C24=N7 bond lengths are 1.3438 (19) and 1.3355 (19) Å, respectively, which confirms that significant double-bond character is present, and that the ligand is in its deprotonated form. Spectroscopic and mass spectrometry analyses of the complex further confirm that the 2:1 deprotonated ligand–metal complex is present.

## Synthesis and crystallization

The title Ni^II^ complex was synthesized by dissolving quinoline-2-carboxaldehyde 4-ethyl­thio­semicarbazone (0.050 g, 0.194 mmol) in hot aceto­nitrile (10 ml), which was mixed with a hot solution of nickel(II) acetate tetra­hydrate (48.2 mg, 0.194 mmol) in methanol (10 ml) on a steam bath, and left to reflux at 355 K for 40 min. On standing overnight at room temperature, dark-brown crystals suitable for X-ray diffraction were obtained [yield: 0.0164 g; m.p. 541 K (decomposition)]. Elemental analysis calculated (%) for C_26.33_H_28.65_N_8_NiOS_2_: C 53.06, H 4.85, N 18.8; found: C 53.22, H 4.75, N 19.14. IR (ν, cm^−1^): 3280, 3217 (N—H), 2965, 2921 (CH ar­yl), 1600 (C=N), 1530, 1469 (C=C arom.), 831 (C—S). UV–Vis (DMSO), λ_max_: 118, 389, 474 nm. HR ESI–MS: calculated for [*M* + H]^+^: 573.1165; found 573.1087 (*M* is the unsolvated complex).

## Refinement

Crystal data, data collection and structure refinement details are summarized in Table 2 ▸. One ethyl group (C25—C26) was modelled for disorder over two parts. Displacement parameters for this group were restrained to be similar and site occupancies were refined to 0.58 (2) and 0.42 (2). The last residual peaks in the difference maps were modelled as a mixture of water and methanol sharing a single site, and displacement parameters for these atoms (C1*A*, O1*A* and O1*W*) were constrained to be identical. Occupancies for water and methanol were refined to 0.672 (6) and 0.328 (6), respectively. The H atoms of the solvent mol­ecules could not be located from a difference map and were thus placed in calculated positions. The H atoms of the amine groups (H4 and H8) were refined freely (positions and isotropic displacement parameters), while other H atoms were placed in calculated positions.

## Supplementary Material

Crystal structure: contains datablock(s) I. DOI: 10.1107/S2414314624003390/bh4083sup1.cif

Structure factors: contains datablock(s) I. DOI: 10.1107/S2414314624003390/bh4083Isup2.hkl

CCDC reference: 2331984

Additional supporting information:  crystallographic information; 3D view; checkCIF report

## Figures and Tables

**Table 1:** Hydrogen-bond geometry (Å, °)

*D*—H⋯*A*	*D*—H	H⋯*A*	*D*⋯*A*	*D*—H⋯*A*
N4—H4⋯O1*W*	0.84 (2)	2.10 (3)	2.936 (12)	174 (2)
N4—H4⋯O1*S*	0.84 (2)	2.08 (4)	2.91 (3)	170 (2)
O1*S*—H1*S*⋯S2^i^	0.84	2.46	3.24 (2)	154
N8—H8⋯N3^ii^	0.85 (2)	2.29 (2)	3.1349 (19)	174 (2)

Symmetry codes: (i) 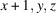 ; (ii) 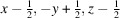 .

**Table 2:** Experimental details

Crystal data	
Chemical formula	[Ni(C_13_H_13_N_4_S)_2_]·0.33CH_4_O·0.67H_2_O
*M*_r_	595.97
Crystal system, space group	Monoclinic, *P*2_1_/*n*
Temperature (K)	100
*a*, *b*, *c* (Å)	10.0560 (6), 21.3696 (9), 12.5648 (6)
β (°)	100.161 (2)
*V* (Å^3^)	2657.7 (2)
*Z*	4
Radiation type	Mo *K*α
μ (mm^−1^)	0.93
Crystal size (mm)	0.13 × 0.13 × 0.10

Data collection	
Diffractometer	Bruker D8 Venture
Absorption correction	Multi-scan (*SADABS*; Krause *et al.*, 2015 ▸)
*T*_min_, *T*_max_	0.710, 0.746
No. of measured, independent and observed [*I* > 2σ(*I*)] reflections	143112, 8094, 7523
*R*_int_	0.076
(sin θ/λ)_max_ (Å^−1^)	0.714

Refinement	
*R*[*F* ^2^ > 2σ(*F* ^2^)], *wR*(*F* ^2^), *S*	0.036, 0.083, 1.08
No. of reflections	8094
No. of parameters	385
No. of restraints	36
H-atom treatment	H atoms treated by a mixture of independent and constrained refinement
Δρ_max_, Δρ_min_ (e Å^−3^)	0.46, −0.48

Computer programs: *APEX4* and *SAINT* (Bruker, 2016 ▸), *SHELXT2018* (Sheldrick, 2015*a* ▸), *SHELXL2019* (Sheldrick, 2015*b* ▸), *Mercury* (Macrae *et al.*, 2020 ▸) and *publCIF* (Westrip, 2010 ▸).

## full crystallographic data

### Crystal data

**Table:**

[Ni(C_13_H_13_N_4_S)_2_]·0.328CH_4_O·0.672H_2_O	*D*_x_ = 1.489 Mg m^−^^3^
*M**_r_* = 595.97	Melting point: 541 K
Monoclinic, *P*2_1_/*n*	Mo *K*α radiation, λ = 0.71073 Å
*a* = 10.0560 (6) Å	Cell parameters from 9658 reflections
*b* = 21.3696 (9) Å	θ = 2.6–30.4°
*c* = 12.5648 (6) Å	µ = 0.93 mm^−^^1^
β = 100.161 (2)°	*T* = 100 K
*V* = 2657.7 (2) Å^3^	Rod, dark brown
*Z* = 4	0.13 × 0.13 × 0.10 mm
*F*(000) = 1242	

### Data collection

**Table:**

Bruker D8 Venture diffractometer	7523 reflections with *I* > 2σ(*I*)
Radiation source: Microfocus Sealed Tube	*R*_int_ = 0.076
φ and ω scans	θ_max_ = 30.5°, θ_min_ = 3.0°
Absorption correction: multi-scan (SADABS; Krause et al., 2015)	*h* = −14→14
*T*_min_ = 0.710, *T*_max_ = 0.746	*k* = −30→30
143112 measured reflections	*l* = −17→17
8094 independent reflections	

### Refinement

**Table:**

Refinement on *F*^2^	Primary atom site location: dual
Least-squares matrix: full	Secondary atom site location: difference Fourier map
*R*[*F*^2^ > 2σ(*F*^2^)] = 0.036	Hydrogen site location: mixed
*wR*(*F*^2^) = 0.083	H atoms treated by a mixture of independent and constrained refinement
*S* = 1.08	*w* = 1/[σ^2^(*F*_o_^2^) + (0.0226*P*)^2^ + 3.305*P*] where *P* = (*F*_o_^2^ + 2*F*_c_^2^)/3
8094 reflections	(Δ/σ)_max_ = 0.002
385 parameters	Δρ_max_ = 0.46 e Å^−^^3^
36 restraints	Δρ_min_ = −0.47 e Å^−^^3^

### Fractional atomic coordinates and isotropic or equivalent isotropic displacement parameters (Å^2^)

**Table:**

	*x*	*y*	*z*	*U*_iso_*/*U*_eq_	Occ. (<1)
Ni1	0.37125 (2)	0.34643 (2)	0.54815 (2)	0.01053 (5)	
S1	0.58288 (4)	0.34892 (2)	0.48937 (3)	0.01341 (8)	
S2	0.23953 (4)	0.30891 (2)	0.37971 (3)	0.01339 (8)	
N1	0.21681 (13)	0.31495 (6)	0.63795 (11)	0.0125 (2)	
N2	0.44276 (13)	0.26050 (6)	0.60052 (10)	0.0113 (2)	
N3	0.55983 (13)	0.23412 (6)	0.58182 (11)	0.0126 (2)	
N4	0.74864 (14)	0.25342 (7)	0.50791 (12)	0.0156 (3)	
H4	0.791 (2)	0.2775 (11)	0.4735 (19)	0.024 (6)*	
N5	0.44660 (13)	0.41468 (6)	0.67711 (10)	0.0119 (2)	
N6	0.31246 (13)	0.42947 (6)	0.47570 (10)	0.0117 (2)	
N7	0.24729 (13)	0.43707 (6)	0.37230 (11)	0.0130 (2)	
N8	0.14719 (14)	0.38603 (7)	0.21967 (11)	0.0159 (3)	
H8	0.125 (2)	0.3518 (11)	0.187 (2)	0.025 (6)*	
C1	0.10008 (15)	0.34256 (7)	0.65722 (13)	0.0143 (3)	
C2	0.07119 (17)	0.40507 (8)	0.62318 (15)	0.0202 (3)	
H2	0.132819	0.427501	0.588242	0.024*	
C3	−0.04576 (18)	0.43326 (9)	0.64064 (17)	0.0244 (4)	
H3	−0.064523	0.475099	0.617169	0.029*	
C4	−0.13860 (17)	0.40102 (9)	0.69295 (16)	0.0220 (3)	
H4A	−0.219476	0.421041	0.703837	0.026*	
C5	−0.11204 (16)	0.34105 (8)	0.72788 (14)	0.0188 (3)	
H5	−0.174306	0.319666	0.763675	0.023*	
C6	0.00747 (15)	0.31046 (8)	0.71120 (13)	0.0148 (3)	
C7	0.03928 (16)	0.24885 (8)	0.74760 (13)	0.0161 (3)	
H7	−0.020396	0.226208	0.784164	0.019*	
C8	0.15664 (16)	0.22215 (7)	0.72970 (13)	0.0146 (3)	
H8A	0.180217	0.181002	0.754807	0.018*	
C9	0.24290 (15)	0.25634 (7)	0.67338 (12)	0.0124 (3)	
C10	0.36676 (15)	0.22762 (7)	0.65212 (12)	0.0124 (3)	
H10	0.390954	0.186202	0.675312	0.015*	
C11	0.63063 (15)	0.27363 (7)	0.53007 (12)	0.0124 (3)	
C12	0.80517 (16)	0.19107 (8)	0.53084 (14)	0.0172 (3)	
H12A	0.786655	0.177009	0.601868	0.021*	
H12B	0.904409	0.193044	0.535614	0.021*	
C13	0.74744 (19)	0.14358 (8)	0.44520 (16)	0.0228 (3)	
H13A	0.790999	0.102944	0.462710	0.034*	
H13B	0.764093	0.157600	0.374460	0.034*	
H13C	0.649940	0.139539	0.443148	0.034*	
C14	0.51401 (15)	0.40752 (7)	0.78126 (12)	0.0123 (3)	
C15	0.54723 (16)	0.34674 (7)	0.82233 (13)	0.0147 (3)	
H15	0.524935	0.311347	0.776942	0.018*	
C16	0.61140 (16)	0.33856 (8)	0.92725 (13)	0.0164 (3)	
H16	0.632372	0.297488	0.953940	0.020*	
C17	0.64651 (17)	0.39034 (8)	0.99577 (14)	0.0181 (3)	
H17	0.689126	0.383985	1.068662	0.022*	
C18	0.61934 (17)	0.44984 (8)	0.95752 (13)	0.0173 (3)	
H18	0.645279	0.484621	1.003611	0.021*	
C19	0.55302 (16)	0.45990 (7)	0.85004 (13)	0.0142 (3)	
C20	0.52339 (17)	0.52050 (8)	0.80729 (13)	0.0171 (3)	
H20	0.548570	0.556342	0.850925	0.020*	
C21	0.45851 (17)	0.52724 (7)	0.70315 (13)	0.0163 (3)	
H21	0.438725	0.567711	0.673239	0.020*	
C22	0.42098 (15)	0.47294 (7)	0.64009 (12)	0.0128 (3)	
C23	0.35131 (16)	0.47991 (7)	0.52932 (13)	0.0136 (3)	
H23	0.334804	0.520070	0.497294	0.016*	
C24	0.21181 (15)	0.38279 (7)	0.32265 (12)	0.0121 (3)	
C25	0.1078 (15)	0.4456 (8)	0.1591 (14)	0.021 (2)	0.58 (2)
H25A	0.161021	0.480574	0.196717	0.025*	0.58 (2)
H25B	0.130426	0.442171	0.085764	0.025*	0.58 (2)
C26	−0.0373 (7)	0.4598 (3)	0.1497 (11)	0.0455 (18)	0.58 (2)
H26A	−0.055776	0.501394	0.117810	0.068*	0.58 (2)
H26B	−0.062309	0.458858	0.221588	0.068*	0.58 (2)
H26C	−0.090205	0.428441	0.103356	0.068*	0.58 (2)
C25A	0.1242 (19)	0.4421 (11)	0.1610 (18)	0.017 (2)	0.42 (2)
H25C	0.181819	0.443287	0.104807	0.021*	0.42 (2)
H25D	0.149015	0.478010	0.210232	0.021*	0.42 (2)
C26A	−0.0277 (10)	0.4477 (6)	0.1064 (12)	0.041 (2)	0.42 (2)
H26D	−0.043953	0.489086	0.073039	0.061*	0.42 (2)
H26E	−0.085129	0.442191	0.161080	0.061*	0.42 (2)
H26F	−0.049072	0.415319	0.050730	0.061*	0.42 (2)
O1W	0.9174 (12)	0.3352 (4)	0.3991 (10)	0.0262 (8)	0.672 (6)
H1W	0.926373	0.363814	0.449173	0.039*	0.672 (6)
H2W	0.999607	0.326761	0.391031	0.039*	0.672 (6)
O1S	0.924 (3)	0.3269 (10)	0.394 (2)	0.0262 (8)	0.328 (6)
H1S	1.007535	0.322428	0.413664	0.039*	0.328 (6)
C1S	0.8922 (6)	0.3926 (3)	0.3797 (5)	0.0262 (8)	0.328 (6)
H1A	0.828778	0.404763	0.426964	0.039*	0.328 (6)
H1B	0.851011	0.400377	0.304168	0.039*	0.328 (6)
H1C	0.975225	0.417242	0.398023	0.039*	0.328 (6)

### Atomic displacement parameters (Å^2^)

**Table:**

	*U*^11^	*U*^22^	*U*^33^	*U*^12^	*U*^13^	*U*^23^
Ni1	0.00948 (9)	0.00951 (9)	0.01260 (9)	0.00096 (6)	0.00196 (7)	0.00092 (7)
S1	0.01215 (16)	0.01128 (16)	0.01730 (18)	0.00052 (12)	0.00392 (13)	0.00194 (13)
S2	0.01316 (16)	0.01044 (15)	0.01588 (17)	0.00003 (12)	0.00072 (13)	0.00007 (13)
N1	0.0099 (5)	0.0132 (6)	0.0148 (6)	0.0006 (4)	0.0033 (5)	0.0001 (5)
N2	0.0094 (5)	0.0121 (5)	0.0122 (6)	0.0006 (4)	0.0012 (4)	−0.0004 (4)
N3	0.0100 (5)	0.0128 (6)	0.0152 (6)	0.0021 (4)	0.0031 (5)	0.0008 (5)
N4	0.0120 (6)	0.0154 (6)	0.0202 (7)	0.0020 (5)	0.0054 (5)	0.0030 (5)
N5	0.0104 (5)	0.0125 (6)	0.0130 (6)	−0.0002 (4)	0.0025 (4)	0.0002 (4)
N6	0.0100 (5)	0.0131 (6)	0.0122 (6)	0.0018 (4)	0.0022 (4)	0.0007 (4)
N7	0.0137 (6)	0.0126 (6)	0.0122 (6)	0.0010 (4)	0.0004 (5)	0.0006 (5)
N8	0.0180 (6)	0.0141 (6)	0.0142 (6)	−0.0001 (5)	−0.0012 (5)	−0.0008 (5)
C1	0.0117 (6)	0.0154 (7)	0.0155 (7)	0.0011 (5)	0.0018 (5)	−0.0005 (5)
C2	0.0163 (7)	0.0166 (7)	0.0297 (9)	0.0037 (6)	0.0094 (7)	0.0032 (6)
C3	0.0182 (8)	0.0192 (8)	0.0377 (10)	0.0053 (6)	0.0104 (7)	0.0014 (7)
C4	0.0127 (7)	0.0245 (8)	0.0298 (9)	0.0040 (6)	0.0067 (6)	−0.0035 (7)
C5	0.0114 (7)	0.0242 (8)	0.0219 (8)	−0.0004 (6)	0.0062 (6)	−0.0036 (6)
C6	0.0103 (6)	0.0195 (7)	0.0148 (7)	−0.0008 (5)	0.0025 (5)	−0.0015 (6)
C7	0.0140 (7)	0.0202 (7)	0.0147 (7)	−0.0026 (6)	0.0040 (5)	0.0010 (6)
C8	0.0138 (7)	0.0152 (7)	0.0148 (7)	−0.0019 (5)	0.0026 (5)	0.0023 (5)
C9	0.0127 (6)	0.0131 (6)	0.0113 (6)	−0.0006 (5)	0.0017 (5)	0.0000 (5)
C10	0.0122 (6)	0.0107 (6)	0.0144 (7)	0.0005 (5)	0.0024 (5)	0.0010 (5)
C11	0.0112 (6)	0.0133 (6)	0.0124 (6)	0.0006 (5)	0.0015 (5)	0.0000 (5)
C12	0.0131 (7)	0.0189 (7)	0.0198 (8)	0.0066 (6)	0.0037 (6)	0.0035 (6)
C13	0.0232 (8)	0.0183 (8)	0.0269 (9)	0.0053 (6)	0.0041 (7)	0.0009 (7)
C14	0.0098 (6)	0.0141 (6)	0.0133 (7)	−0.0001 (5)	0.0024 (5)	0.0002 (5)
C15	0.0155 (7)	0.0136 (7)	0.0146 (7)	0.0011 (5)	0.0017 (5)	−0.0003 (5)
C16	0.0159 (7)	0.0159 (7)	0.0168 (7)	0.0021 (5)	0.0015 (6)	0.0020 (6)
C17	0.0171 (7)	0.0202 (7)	0.0155 (7)	0.0005 (6)	−0.0012 (6)	0.0001 (6)
C18	0.0183 (7)	0.0170 (7)	0.0153 (7)	−0.0018 (6)	−0.0009 (6)	−0.0024 (6)
C19	0.0137 (7)	0.0143 (7)	0.0145 (7)	−0.0012 (5)	0.0020 (5)	−0.0006 (5)
C20	0.0209 (8)	0.0134 (7)	0.0160 (7)	−0.0019 (6)	0.0009 (6)	−0.0020 (6)
C21	0.0201 (7)	0.0117 (6)	0.0170 (7)	−0.0008 (5)	0.0030 (6)	−0.0004 (5)
C22	0.0119 (6)	0.0124 (6)	0.0140 (7)	−0.0002 (5)	0.0026 (5)	0.0000 (5)
C23	0.0144 (7)	0.0115 (6)	0.0146 (7)	0.0010 (5)	0.0021 (5)	0.0019 (5)
C24	0.0093 (6)	0.0132 (6)	0.0138 (7)	0.0006 (5)	0.0022 (5)	0.0006 (5)
C25	0.030 (5)	0.014 (2)	0.017 (2)	0.004 (3)	−0.002 (3)	0.0074 (18)
C26	0.028 (2)	0.031 (2)	0.068 (4)	0.0062 (17)	−0.017 (3)	0.003 (3)
C25A	0.010 (3)	0.022 (4)	0.016 (3)	0.001 (3)	−0.006 (2)	−0.003 (3)
C26A	0.030 (3)	0.037 (4)	0.049 (5)	0.002 (3)	−0.011 (3)	0.020 (3)
O1W	0.0180 (11)	0.033 (2)	0.0285 (12)	−0.0032 (15)	0.0077 (8)	−0.0046 (16)
O1S	0.0180 (11)	0.033 (2)	0.0285 (12)	−0.0032 (15)	0.0077 (8)	−0.0046 (16)
C1S	0.0180 (11)	0.033 (2)	0.0285 (12)	−0.0032 (15)	0.0077 (8)	−0.0046 (16)

### Geometric parameters (Å, º)

**Table:**

Ni1—N6	2.0339 (13)	C12—C13	1.518 (2)
Ni1—N2	2.0384 (13)	C12—H12A	0.9900
Ni1—N1	2.1808 (13)	C12—H12B	0.9900
Ni1—N5	2.2120 (13)	C13—H13A	0.9800
Ni1—S1	2.3731 (4)	C13—H13B	0.9800
Ni1—S2	2.4264 (4)	C13—H13C	0.9800
S1—C11	1.7303 (15)	C14—C15	1.416 (2)
S2—C24	1.7359 (15)	C14—C19	1.426 (2)
N1—C9	1.3395 (19)	C15—C16	1.373 (2)
N1—C1	1.3730 (19)	C15—H15	0.9500
N2—C10	1.2935 (19)	C16—C17	1.408 (2)
N2—N3	1.3625 (17)	C16—H16	0.9500
N3—C11	1.3438 (19)	C17—C18	1.370 (2)
N4—C11	1.3376 (19)	C17—H17	0.9500
N4—C12	1.457 (2)	C18—C19	1.413 (2)
N4—H4	0.84 (2)	C18—H18	0.9500
N5—C22	1.3381 (19)	C19—C20	1.413 (2)
N5—C14	1.3720 (19)	C20—C21	1.363 (2)
N6—C23	1.294 (2)	C20—H20	0.9500
N6—N7	1.3578 (18)	C21—C22	1.418 (2)
N7—C24	1.3355 (19)	C21—H21	0.9500
N8—C24	1.343 (2)	C22—C23	1.451 (2)
N8—C25A	1.40 (2)	C23—H23	0.9500
N8—C25	1.499 (15)	C25—C26	1.474 (15)
N8—H8	0.85 (2)	C25—H25A	0.9900
C1—C2	1.417 (2)	C25—H25B	0.9900
C1—C6	1.422 (2)	C26—H26A	0.9800
C2—C3	1.373 (2)	C26—H26B	0.9800
C2—H2	0.9500	C26—H26C	0.9800
C3—C4	1.412 (3)	C25A—C26A	1.56 (2)
C3—H3	0.9500	C25A—H25C	0.9900
C4—C5	1.366 (3)	C25A—H25D	0.9900
C4—H4A	0.9500	C26A—H26D	0.9800
C5—C6	1.416 (2)	C26A—H26E	0.9800
C5—H5	0.9500	C26A—H26F	0.9800
C6—C7	1.412 (2)	O1W—H1W	0.8700
C7—C8	1.365 (2)	O1W—H2W	0.8700
C7—H7	0.9500	O1S—C1S	1.44 (2)
C8—C9	1.416 (2)	O1S—H1S	0.8400
C8—H8A	0.9500	C1S—H1A	0.9800
C9—C10	1.455 (2)	C1S—H1B	0.9800
C10—H10	0.9500	C1S—H1C	0.9800
			
N6—Ni1—N2	171.31 (5)	N4—C12—H12B	109.1
N6—Ni1—N1	108.87 (5)	C13—C12—H12B	109.1
N2—Ni1—N1	78.34 (5)	H12A—C12—H12B	107.8
N6—Ni1—N5	77.74 (5)	C12—C13—H13A	109.5
N2—Ni1—N5	107.56 (5)	C12—C13—H13B	109.5
N1—Ni1—N5	90.43 (5)	H13A—C13—H13B	109.5
N6—Ni1—S1	92.69 (4)	C12—C13—H13C	109.5
N2—Ni1—S1	80.56 (4)	H13A—C13—H13C	109.5
N1—Ni1—S1	158.05 (4)	H13B—C13—H13C	109.5
N5—Ni1—S1	90.18 (4)	N5—C14—C15	119.67 (14)
N6—Ni1—S2	80.04 (4)	N5—C14—C19	121.82 (14)
N2—Ni1—S2	95.23 (4)	C15—C14—C19	118.50 (14)
N1—Ni1—S2	91.16 (4)	C16—C15—C14	120.50 (15)
N5—Ni1—S2	157.00 (4)	C16—C15—H15	119.7
S1—Ni1—S2	96.747 (15)	C14—C15—H15	119.7
C11—S1—Ni1	96.00 (5)	C15—C16—C17	120.78 (15)
C24—S2—Ni1	94.75 (5)	C15—C16—H16	119.6
C9—N1—C1	117.82 (13)	C17—C16—H16	119.6
C9—N1—Ni1	110.17 (10)	C18—C17—C16	120.13 (15)
C1—N1—Ni1	131.89 (11)	C18—C17—H17	119.9
C10—N2—N3	117.81 (13)	C16—C17—H17	119.9
C10—N2—Ni1	116.44 (10)	C17—C18—C19	120.52 (15)
N3—N2—Ni1	125.69 (10)	C17—C18—H18	119.7
C11—N3—N2	111.78 (12)	C19—C18—H18	119.7
C11—N4—C12	125.70 (14)	C18—C19—C20	122.34 (15)
C11—N4—H4	117.6 (16)	C18—C19—C14	119.52 (14)
C12—N4—H4	116.6 (16)	C20—C19—C14	118.15 (14)
C22—N5—C14	117.88 (13)	C21—C20—C19	119.66 (15)
C22—N5—Ni1	109.80 (10)	C21—C20—H20	120.2
C14—N5—Ni1	132.30 (10)	C19—C20—H20	120.2
C23—N6—N7	116.66 (13)	C20—C21—C22	119.02 (15)
C23—N6—Ni1	117.15 (10)	C20—C21—H21	120.5
N7—N6—Ni1	125.86 (10)	C22—C21—H21	120.5
C24—N7—N6	112.77 (12)	N5—C22—C21	123.45 (14)
C24—N8—C25A	123.8 (9)	N5—C22—C23	117.36 (13)
C24—N8—C25	124.9 (7)	C21—C22—C23	119.19 (14)
C24—N8—H8	117.5 (16)	N6—C23—C22	117.65 (14)
C25A—N8—H8	118.5 (19)	N6—C23—H23	121.2
C25—N8—H8	117.6 (17)	C22—C23—H23	121.2
N1—C1—C2	119.21 (14)	N7—C24—N8	116.68 (14)
N1—C1—C6	121.95 (14)	N7—C24—S2	125.94 (12)
C2—C1—C6	118.83 (14)	N8—C24—S2	117.37 (12)
C3—C2—C1	120.07 (16)	C26—C25—N8	112.6 (10)
C3—C2—H2	120.0	C26—C25—H25A	109.1
C1—C2—H2	120.0	N8—C25—H25A	109.1
C2—C3—C4	121.03 (17)	C26—C25—H25B	109.1
C2—C3—H3	119.5	N8—C25—H25B	109.1
C4—C3—H3	119.5	H25A—C25—H25B	107.8
C5—C4—C3	119.96 (16)	C25—C26—H26A	109.5
C5—C4—H4A	120.0	C25—C26—H26B	109.5
C3—C4—H4A	120.0	H26A—C26—H26B	109.5
C4—C5—C6	120.58 (16)	C25—C26—H26C	109.5
C4—C5—H5	119.7	H26A—C26—H26C	109.5
C6—C5—H5	119.7	H26B—C26—H26C	109.5
C7—C6—C5	122.16 (15)	N8—C25A—C26A	110.7 (14)
C7—C6—C1	118.33 (14)	N8—C25A—H25C	109.5
C5—C6—C1	119.50 (15)	C26A—C25A—H25C	109.5
C8—C7—C6	119.31 (15)	N8—C25A—H25D	109.5
C8—C7—H7	120.3	C26A—C25A—H25D	109.5
C6—C7—H7	120.3	H25C—C25A—H25D	108.1
C7—C8—C9	119.38 (15)	C25A—C26A—H26D	109.5
C7—C8—H8A	120.3	C25A—C26A—H26E	109.5
C9—C8—H8A	120.3	H26D—C26A—H26E	109.5
N1—C9—C8	123.19 (14)	C25A—C26A—H26F	109.5
N1—C9—C10	117.21 (13)	H26D—C26A—H26F	109.5
C8—C9—C10	119.61 (14)	H26E—C26A—H26F	109.5
N2—C10—C9	117.62 (13)	H1W—O1W—H2W	104.5
N2—C10—H10	121.2	C1S—O1S—H1S	109.5
C9—C10—H10	121.2	O1S—C1S—H1A	109.5
N4—C11—N3	117.59 (14)	O1S—C1S—H1B	109.5
N4—C11—S1	116.67 (12)	H1A—C1S—H1B	109.5
N3—C11—S1	125.74 (12)	O1S—C1S—H1C	109.5
N4—C12—C13	112.58 (14)	H1A—C1S—H1C	109.5
N4—C12—H12A	109.1	H1B—C1S—H1C	109.5
C13—C12—H12A	109.1		
			
C10—N2—N3—C11	−178.48 (14)	C22—N5—C14—C15	−177.83 (14)
Ni1—N2—N3—C11	4.53 (18)	Ni1—N5—C14—C15	0.2 (2)
C23—N6—N7—C24	179.47 (13)	C22—N5—C14—C19	1.7 (2)
Ni1—N6—N7—C24	6.23 (18)	Ni1—N5—C14—C19	179.77 (11)
C9—N1—C1—C2	178.43 (15)	N5—C14—C15—C16	−178.22 (15)
Ni1—N1—C1—C2	−6.1 (2)	C19—C14—C15—C16	2.2 (2)
C9—N1—C1—C6	−1.1 (2)	C14—C15—C16—C17	−0.6 (2)
Ni1—N1—C1—C6	174.43 (11)	C15—C16—C17—C18	−1.4 (3)
N1—C1—C2—C3	179.27 (17)	C16—C17—C18—C19	1.6 (3)
C6—C1—C2—C3	−1.2 (3)	C17—C18—C19—C20	−179.83 (16)
C1—C2—C3—C4	0.4 (3)	C17—C18—C19—C14	0.1 (2)
C2—C3—C4—C5	0.6 (3)	N5—C14—C19—C18	178.44 (14)
C3—C4—C5—C6	−0.6 (3)	C15—C14—C19—C18	−2.0 (2)
C4—C5—C6—C7	179.03 (16)	N5—C14—C19—C20	−1.6 (2)
C4—C5—C6—C1	−0.2 (3)	C15—C14—C19—C20	177.97 (15)
N1—C1—C6—C7	1.3 (2)	C18—C19—C20—C21	−179.63 (16)
C2—C1—C6—C7	−178.14 (15)	C14—C19—C20—C21	0.4 (2)
N1—C1—C6—C5	−179.35 (15)	C19—C20—C21—C22	0.6 (2)
C2—C1—C6—C5	1.2 (2)	C14—N5—C22—C21	−0.7 (2)
C5—C6—C7—C8	−179.50 (16)	Ni1—N5—C22—C21	−179.18 (12)
C1—C6—C7—C8	−0.2 (2)	C14—N5—C22—C23	179.15 (13)
C6—C7—C8—C9	−1.1 (2)	Ni1—N5—C22—C23	0.68 (17)
C1—N1—C9—C8	−0.3 (2)	C20—C21—C22—N5	−0.4 (2)
Ni1—N1—C9—C8	−176.77 (12)	C20—C21—C22—C23	179.71 (15)
C1—N1—C9—C10	179.83 (13)	N7—N6—C23—C22	179.89 (13)
Ni1—N1—C9—C10	3.41 (16)	Ni1—N6—C23—C22	−6.25 (18)
C7—C8—C9—N1	1.4 (2)	N5—C22—C23—N6	3.5 (2)
C7—C8—C9—C10	−178.73 (14)	C21—C22—C23—N6	−176.61 (14)
N3—N2—C10—C9	179.06 (13)	N6—N7—C24—N8	−179.73 (13)
Ni1—N2—C10—C9	−3.66 (18)	N6—N7—C24—S2	1.23 (19)
N1—C9—C10—N2	−0.1 (2)	C25A—N8—C24—N7	4.5 (11)
C8—C9—C10—N2	−179.93 (14)	C25—N8—C24—N7	−2.6 (8)
C12—N4—C11—N3	2.8 (2)	C25A—N8—C24—S2	−176.4 (11)
C12—N4—C11—S1	−177.32 (13)	C25—N8—C24—S2	176.5 (8)
N2—N3—C11—N4	179.34 (13)	Ni1—S2—C24—N7	−5.77 (14)
N2—N3—C11—S1	−0.52 (19)	Ni1—S2—C24—N8	175.20 (12)
Ni1—S1—C11—N4	177.62 (12)	C24—N8—C25—C26	−103.1 (11)
Ni1—S1—C11—N3	−2.52 (14)	C24—N8—C25A—C26A	−129.9 (12)
C11—N4—C12—C13	82.1 (2)		

### Hydrogen-bond geometry (Å, º)

**Table:**

*D*—H···*A*	*D*—H	H···*A*	*D*···*A*	*D*—H···*A*
N4—H4···O1*W*	0.84 (2)	2.10 (3)	2.936 (12)	174 (2)
N4—H4···O1*S*	0.84 (2)	2.08 (4)	2.91 (3)	170 (2)
O1*S*—H1*S*···S2^i^	0.84	2.46	3.24 (2)	154
N8—H8···N3^ii^	0.85 (2)	2.29 (2)	3.1349 (19)	174 (2)

Symmetry codes: (i) *x*+1, *y*, *z*; (ii) *x*−1/2, −*y*+1/2, *z*−1/2.
